# A Type II Arabinogalactan from *Anoectochilus formosanus* for G-CSF Production in Macrophages and Leukopenia Improvement in CT26-Bearing Mice Treated with 5-Fluorouracil

**DOI:** 10.1155/2013/458075

**Published:** 2013-09-26

**Authors:** Li-Chan Yang, Ting-Jang Lu, Wen-Chuan Lin

**Affiliations:** ^1^Institute of Food Science and Technology, National Taiwan University, Taipei, Taiwan; ^2^School of Medicine, Graduate Institute of Basic Medical Science and Tsuzuki Institute for Traditional Medicine, China Medical University, No. 91 Hsueh Shih Road, Taichung 40402, Taiwan

## Abstract

*Anoectochilus formosanus* is an herb well known in Asian countries. The polysaccharide isolated from *A. formosanus* consists of type II arabinogalactan (AGAF), with branched 3,6-Gal as the major moiety. In this study, AGAF was examined for the granulocyte colony-stimulating factor (G-CSF) production and related protein expression in RAW 264.7 murine macrophages. The signaling pathway of G-CSF production involves AGAF and mitogen-activated protein kinases (MAPKs) inhibitors and pattern-recognition receptor antibodies. AGAF was evaluated to ease the leukopenia in CT26-colon-cancer-bearing mice treated with 5-fluorouracil (5-FU). The results of this study showed that AGAF was a stimulant for Toll-like receptor 2 and Dectin-1 and that it induced G-CSF production, through p38 and ERK MAPK, and NF-**κ**B pathways. In vivo examination showed that the oral administration of AGAF mitigated the side effects of leukopenia caused by 5-FU in colon-cancer-bearing mice. In conclusion, the botanic type II AGAF in this study was a potent G-CSF inducer in vivo and in vitro.

## 1. Introduction

*Anoectochilus formosanus*, a well-known medicinal orchid, is widely used in Asian countries. *A. formosanus *exhibits hepatoprotective activity [1], antitumor and immunomodulatory effects [2, 3], asthma treatment effects [4], antihyperglycemic activity [5], prebiotic effects [6], and antiosteoporosis activity [7, 8]. The polysaccharide isolated from *A. formosanus* juice is a type II arabinogalactan with an average molecular weight of 29 kDa, and it is a prebiotic that increases the growth of probiotics in vivo and in vitro [6]. 

Functional polysaccharides exist in botanicals, microorganisms, and animals, and they are well-known immunomodulatory agents. Polysaccharides stimulate macrophages for cell proliferation, cytokine production, and phagocytosis [9]. Polysaccharides can stimulate the macrophage production of granulocyte colony-stimulating factor (G-CSF), directly affect neutrophil proliferation, and modulate other immune activities [9].

Under the basal conditions of hematopoiesis, G-CSF is the major regulator of neutrophil production and is also referred to as colony-stimulating factor 3 [10]. G-CSF is a unique colony-stimulating hormone that suppresses the production of proinflammatory cytokines while simultaneously activating the antibacterial defense of neutrophils [11]. G-CSF is not only required for differentiating neutrophils in the bone marrow, but it also elicits potent anti-inflammatory effects in monocytes and in septic mice simultaneously [11–13]. 

Numerous studies have reported that polysaccharides can stimulate the secretion of G-CSF in vivo and in vitro [14–16]. Ito et al. [16] showed that *β*-glucan isolated from *Grifola frondosa* stimulates the production of G-CSF in vivo and in vitro. The *β*-glucan isolated from *G. frondosa* has been investigated for its therapeutic effects and decreases in myelosuppression and nephrotoxicity of cisplatin in mice [17]. The *β*-glucan isolated from *G. frondosa* promotes the recovery of leukocytes and myeloid cell function in peripheral blood from paclitaxel hematotoxicity [18].

Botanic polysaccharides are thought to mediate macrophages through the recognition of polysaccharides by specific surface receptors that are known as pattern-recognition receptors (PRRs), such as Toll-like receptors (TLRs) and Dectin-1 [9]. The polysaccharides with immune-stimulating bioactivity are thought to have structural features as pathogen-associated molecular patterns (PAMPs) and to mediate innate immunity by binding to PRRs [19]. 

Black soybean polysaccharide also promotes myelopoiesis after chemotherapy and irradiation therapy in mice. Current cancer therapies include surgery, chemotherapy, radiation therapy, proton therapy, and targeted cancer therapy. Although chemotherapy and radiation therapy are the most prevalent of these cancer therapies, they cause severe side effects. Cytotoxic chemotherapy suppresses the hematopoietic system, impairing host protective mechanisms and limiting the doses of chemotherapy that can be tolerated [20]. Neutropenia, the most severe hematologic toxicity, is associated with the risk of life-threatening infections, as well as chemotherapy dose reductions and delays that may compromise treatment outcomes [21]. G-CSF can effectively reduce the incidence of febrile neutropenia when administered immediately after chemotherapy [22].

The results of this study showed that AGAF stimulates G-CSF and the possible signaling pathway of G-CSF secretion. In addition, the effect of oral administrated with AGAF was investigated on reducing of leukopenia after chemotherapy in colon cancer bearing mice treated with 5-fluorouracil.

## 2. Materials and Methods 

### 2.1. Preparation of Type II Arabinogalactan from *A. formosanus* (AGAF)

The plant of* A. formosanus *was purchased from Yu-Jung Farm (Puli, Taiwan). This orchid species is not under international protection and conservation. Previous research has described the AGAF preparation process [6]. In brief, tissue-cultured *A. formosanus* was homogenized with distilled water and then partitioned with ethyl acetate. Ethanol was added into the aqueous extracts of *A. formosanus* to precipitate crude polysaccharides, and the crude polysaccharide was then treated with *α*-amylase, protease, and amyloglucosidase (Megazyme, Wicklow, Ireland) to remove starches and proteins. After enzymatic treatment, AGAF was preserved in ethanol until use.

The AGAF in this study consisted of type II arabinogalactan (>80%), which was identified based on precipitation with a *β*-glucosyl Yariv reagent [6]. The AGAF yield rate was 0.15% from fresh plants. Chemical analyses showed that the AGAF contained 95.5% carbohydrates and 1.0% protein. Structural analysis showed that AGAF was a type II arabinogalactan consisting primarily of a (1→3)-*β*-D-galactan backbone with a (1→6)-*β*-D-galactan side chain. The monosaccharide composition of AGAF was arabinose, galactose, glucose, and mannose at a ratio of 22.4 : 56.5 : 15.4 : 5.4.

In the endotoxin assay performed in this study, a ToxinSensorTM chromogenic LAL endotoxin assay kit (GenScript, NJ, USA) was used to evaluate AGAF for possible lipopolysaccharide (LPS) contamination. The endotoxin assay results showed that *Escherichia coli *endotoxin standards exhibited a linear relationship between concentrations and optical density values at 545 nm. According to the equation of standard curve, the endotoxin contents of 50, 100, and 150 *μ*g/mL AGAF were less than 0.01 EU/mL (0.006, 0.008, and 0.009 EU/mL, resp.).

### 2.2. Cell Culture and Experimental Design

Murine macrophage RAW 264.7 cells were purchased from the Food Industry Research and Development Institute (Hsinchu, Taiwan) and cultured in Dulbecco's modified Eagle's medium (Invitrogen, CA, USA) supplemented with 10% (v/v) of fetal bovine serum (Gibco, CA, USA), 100 *μ*g/mL of streptomycin, and 100 U/mL of penicillin (Gibco, CA, USA) at 37°C in a humidified atmosphere containing 5% CO_2_. A commercial reagent (CellTiter, Promega, WI, USA) was used to measure cell viability under AGAF treatment. The inhibitors used in this experiment, including SB203580 (10 *μ*M), SP600125 (10 *μ*M), PD98059 (10 *μ*M), and pyrrolidinedithiocarbamic acid (PDTC, 25 *μ*M) (Sigma-Aldrich, MO, USA), were added to cells 60 min prior to adding AGAF. The cells were cultured in a medium alone or a medium containing various concentrations of AGAF (50, 100, or 150 *μ*g/mL).

### 2.3. ELISA Analysis

The RAW 264.7 cells used in this study were plated at a density of 3 × 10^4^ cells/well in a final volume of 200 *μ*L in 96-well plates for 24 h. After 24 h, the RAW 264.7 cells were stimulated with or without AGAF (50, 100, or 150 *μ*g/mL) for 0, 6, 8, 12, 16, 20, and 24 h. The SB203580, PP600125, PD98059, and PDTC inhibitors were added to the wells to determine the relationships among MAPK, NF-*κ*B, and the production of G-CSF. After incubation, a Murine G-CSF ELISA Development kit (PEPROTECH, NJ, USA) was used to detect G-CSF in the supernatants.

### 2.4. Western Blot Analysis

The RAW 264.7 cells used in this study were plated at a density of  1 × 10^5^ cell/mL and stimulated with AGAF (at 50, 100, or 150 *μ*g/mL) or a medium. The treatment periods were 15 and 60 min for cytoplasm and nuclei proteins, respectively. After this treatment, cells were lysed with a Nonidet-P40 buffer, following the method described by Natori et al. [23], to release the protein of interest. This buffer contained protease inhibitors. A Bradford reagent (Sigma-Aldrich, MO, USA) was used to measure the protein contents of the cell extracts, with bovine serum albumin as a standard.

Cell extracts were prepared and resolved by 12% SDS-PAGE before being transferred to polyvinylidene fluoride membranes (Millipore, MA, USA). After blocking, the membranes were incubated with primary and secondary antibodies, washed thoroughly, and examined using a Pierce ECL Plus substrate (Thermal, IL, USA). The band densities were scanned and quantified by an ImageQuant LAS 4000 Imager (Fuji Film, Tokyo, Japan). The primary antibodies included anti-ERK1/2 phosphospecific antibody, anti-ERK1/2 antibody, anti-JNK1/2 phosphospecific antibody, anti-JNK1/2 antibody, anti-p38-MAPK phosphospecific antibody, anti-p38-MAPK antibody, anti-cJun antibody, anti-cfos antibody, anti-p65 antibody, anti-p50 antibody (Cell Signaling Technology, Danvers, MA, USA), anti-C/EBP*β* antibody (Santa Cruz Biotechnology, CA, USA), and anti-actin antibody (Millipore, MA, USA). *Horseradish-peroxidase-* (HRP-) linked anti-rabbit IgG antibodies and HRP-linked anti-mouse IgG antibodies (Santa Cruz Biotechnology) were used as secondary antibodies. 

### 2.5. mRNA Extraction and Reverse Transcriptase-PCR Analysis

The RAW 264.7 cells used in this study were plated at a density of 2 × 10^5^ cells/well in 24-well tissue culture plate (NUNC, Roskilde, Denmark). Cells were treated with a medium alone or a medium containing AGAF (50, 100, or 150 *μ*g/mL) or LPS for 6 h. The inhibitors were added to the cells 1 h prior to AGAF and LPS stimulation to determine the relationship among MAPK, NF-*κ*B, and the gene expression of G-CSF. A TRIzol kit (Invitrogen) was used to extract RNA from the cells. A 3 *μ*g sample of RNA was subjected to reverse transcription (RT) with Moloney murine leukemia virus reverse transcriptase (Promega, WI, USA) in a 50 *μ*L reaction volume. Aliquots of the RT mix were used to amplify fragments of G-CSF by performing PCR. The primers used for murine G-CSF were 5′-CACTTCCGAGTTTTGTTCTC-3′ and 5′-TAAACAGGGATGTCTTGTCC-3′ (product size 238 bp), and those for *glyceraldehyde-3-phosphate dehydrogenase *(*GAPDH*) were 5′-TGTGTCCGTCGTGGATCTGA-3′ and 5′-CCTGCTTCACCACCTTCTTGA-3′ (product size 76 bp). The expression levels of all of the transcripts were normalized to that of the *GAPDH *mRNA in the same tissue sample. The PCR products were separated on a 2% agarose gel and recorded on Polaroid film; the bands were quantified using a densitometer.

### 2.6. Analysis of NF-*κ*B Activation

The activation of NF-*κ*B was measured using a luciferase reporter gene assay. The RAW 264.7 cells were planted in a 6 cm dish for 24 h. The medium was then replaced with serum-free Opti-MEM (Gibco, CA, USA). Cells were transfected with the pNF*κ*B-Luc plasmid reporter gene for 24 h of incubation. After incubation, the medium was replaced with a complete medium for 24 h. The cells were then plated in 24-well tissue culture plates for 12 h and treated with AGAF (at 50, 100, or 150 *μ*g/mL) or a medium only for 4 h. Each well was washed twice with a cold PBS buffer, and the cells were harvested in 150 *μ*L of a lysis buffer (0.5 M HEPES, pH 7.8, 1% triton N-101, 1 mM CaCl_2_, and 1 mM MgCl_2_). Cell lysate aliquots of 100 *μ*L were used for luciferase assay by using the Dual-Luciferase Reporter Assay system (Promega, WI, USA). A TRAID LTELISA reader was used to measure luminescence. Luciferase activities were normalized to protein concentrations, which were determined by using the Bradford reagent (Sigma-Aldrich, MO, USA). 

### 2.7. Inhibition of AGAF-Induced G-CSF Production Using Pattern-Recognition Receptor Antibodies

To determine the role of Toll-like receptor 2 (TLR2), Toll-like receptor 4 (TLR4), Dectin-1, and Complement receptor 3 (CR3) in G-CSF production, the RAW 264.7 cells cultivated in 24-well tissue culture plates were pretreated with TLR2-specific mAb mT2.4 (sc-73361, Santa Cruz), TLR4-specific mAb MTS510 (sc-13591, Santa Cruz), Dectin-1-specific mAb 2A11 (GTX41467, GeneTex), or CR3 (CD11b, GTX42473, GeneTex) mAb at a concentration of 10 *μ*g/mL for 1 h. Isotype control antibodies were used at 10 *μ*g/mL for rat IgG2 (sc-2006, Santa Cruz Biotechnology) and mouse IgG (sc-2005, Santa Cruz Biotechnology). The RAW 264.7 cells were then treated with AGAF (at 100 *μ*g/mL) or a medium for 16 h. After incubation, the levels of G-CSF in the supernatants were measured by commercial ELISA kits.

### 2.8. Protective Effects of AGAF in CT26-Colon-Cancer-Bearing Mice under 5-Fluorouracil Treatment

Six-week-old male BALB/c mice were purchased from the National Laboratory Animal Center (Taipei, Taiwan). Each experiment involved using 10 mice. The mice were housed in a humidity- and temperature-controlled environment and given free access to food and water before the experiments. The body weights of the mice were measured every 3 d. 

CT26 carcinoma cells (1 × 10^6^ cells/mouse) were subcutaneously inoculated into the BALB/c mice on Day 0. The mice were orally administrated 15 or 45 mg/kg of AGAF beginning on Day 2. The mice were intraperitoneally injected with 25 mg/kg of 5-FU (Pharmachemie BV, Haarlem, The Netherlands) or regular saline every other day after Day 2. Tumors were measured every 3 days beginning on Day 7. The tumor sizes were calculated according to the following formula: volume (cm^3^) = 0.5 × *A* × *B* ^2^, where *A* is the longest length and *B* is the shortest length [24]. The mice were euthanized under CO_2_ anesthesia on Day 21. The spleens and tumors were immediately removed and weighed. The blood was collected in an EDTA-tube for complete blood count (CBC) tests.

### 2.9. Statistical Methods

The results are expressed in this paper as means ± standard deviation (SD). All experimental data without Figure 7 were analyzed using one-way analysis of variance with Dunnett's test. Values of *P* < 0.05 were considered statistically significant. The experimental data in Figure 7 were analyzed using one-way analysis of variance with Duncan's multiple test. Values of *P* < 0.05 were considered statistically significant. 

## 3. Results

### 3.1. AGAF Induces G-CSF Secretion in a Time- and Dose-Dependent Manner

**Figure 1:**
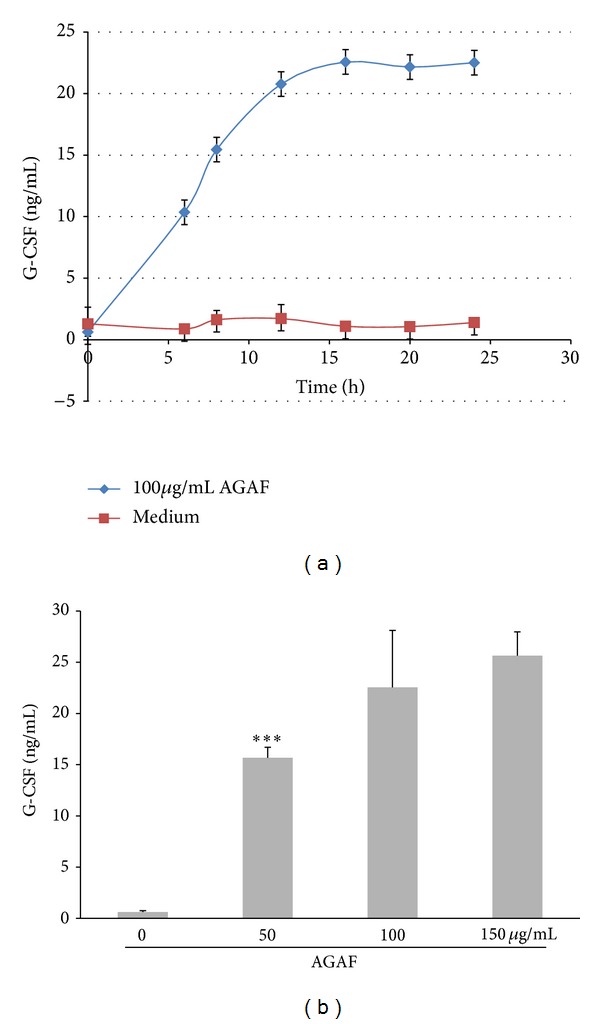
Effect of AGAF on G-CSF expression in RAW 264.7 cells in time-dependent and dose-dependnt manners. (a) Time-dependent manner of G-CSF in RAW 264.7 cells stimulated with 100 *μ*g/mL of AGAF for 0~24 h. (b) Dose-dependent manner of G-CSF in RAW 264.7 cells stimulated with 0, 50, 100, and 150 *μ*g/mL of AGAF for 16 h. The G-CSF level was determined by ELISA. The values are presented as the mean ± SD, *n* = 3. ***P < 0.001 as compared to control group as analyzed by Dunnett's test.

The results of cell viability under AGAF treatment indicate that AGAF did not cause any cytotoxicity in the RAW 264.7 cells (data unpublished). To determine the effects of AGAF on G-CSF secretion, RAW 264.7 cells were incubated with 100 *μ*g/mL of AGAF for 0–24 h, and the G-CSF concentration was measured using ELISA. The G-CSF content increased in a time-dependent manner, initially increasing to 10.4 ng/mL after 6 h of treatment and peaking at 22.5 ng/mL after 16 h (Figure 1(a)). To examine the dose effect of AGAF on G-CSF expression, RAW 264.7 cells were incubated for 16 h with 0–150 *μ*g/mL of AGAF, revealing a dose-dependent rise in G-CSF expression. The G-CSF levels under AGAF (50, 100, and 150 *μ*g/mL) stimulation for 16 h increased by 15.7 ng/mL, 22.5 ng/mL, and 25.6 ng/m, respectively (Figure 1(b)). 

### 3.2. Western Blot Analysis

The possible involvement of signaling pathways in AGAF-induced G-CSF production was explored using several inhibitors, including SB203580 (p38-MAPK inhibitor), SP600125 (JNK1/2 inhibitor), PD98059 (ERK inhibitor), and PDTC (NF-*κ*B activation inhibitor).

**Figure 2:**
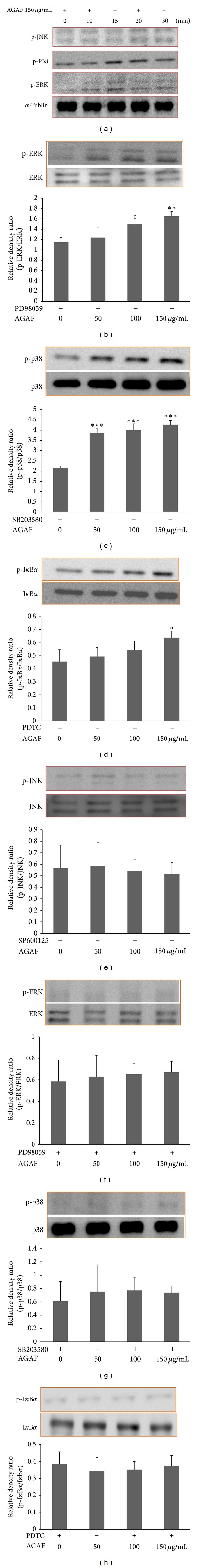
Effect of AGAF on MAPK and I*κ*B*α* phosphorylation in RAW 264.7 cells. (a) AGAF treatment (100 *μ*g/mL) for 0 to 30 min in RAW 264.7 cells. (b–e) The phosphorylation of ERK, p38, I*κ*B*α*, and JNK in RAW 264.7 cells treated with AGAF (50, 100, and 150 *μ*g/mL) or a medium for 15 min. (f) The phosphorylation of ERK in RAW 264.7 cells that were pretreated with 10 *μ*M ERK inhibitor, PD98059, for 60 min and then treated with AGAF (50, 100, and 150 *μ*g/mL) or a medium for 15 min. (g) The phosphorylation of p38 in RAW 264.7 cells that were pretreated with 10 *μ*M p38 inhibitor, SB203580, for 60 min and then treated with AGAF (50, 100, and 150 *μ*g/mL) or a medium for 15 min. (h) The phosphorylation of I*κ*B*α* in RAW 264.7 cells that were pretreated with 25 *μ*M I*κ*B*α* inhibitor, PDTC, for 60 min and then treated with AGAF (50, 100, and 150 *μ*g/mL) or a medium for 15 min. The protein expression was assessed via western blotting. The values are presented as the mean ± SD; *n* = 3. *P < 0.05, **P < 0.01 and ***P < 0.001 as compared to control group as analyzed by Dunnett's test.

As Figure 2(a) showed, ERK1/2 and p38-MAPK were rapidly phosphorylated; the phosphorylation of ERK1/2 and p38-MAPK peaked 15 min after AGAF treatment (100 *μ*g/mL). Western blot analysis showed that AGAF induced the phosphorylation of ERK1/2 (Figure 2(b)), p38-MAPK (Figure 2(c)), and I*κ*B*α* (Figure 2(d)) in RAW264.7 cells, but not the phosphorylation of JNK (Figure 2(e)). The AGAF-induced phosphorylation of ERK1/2, p38-MAPK, and I*κ*B*α* was markedly reduced by PD98059, SB203580, and PDTC, respectively (Figures 2(f)–2(h)).

**Figure 3:**
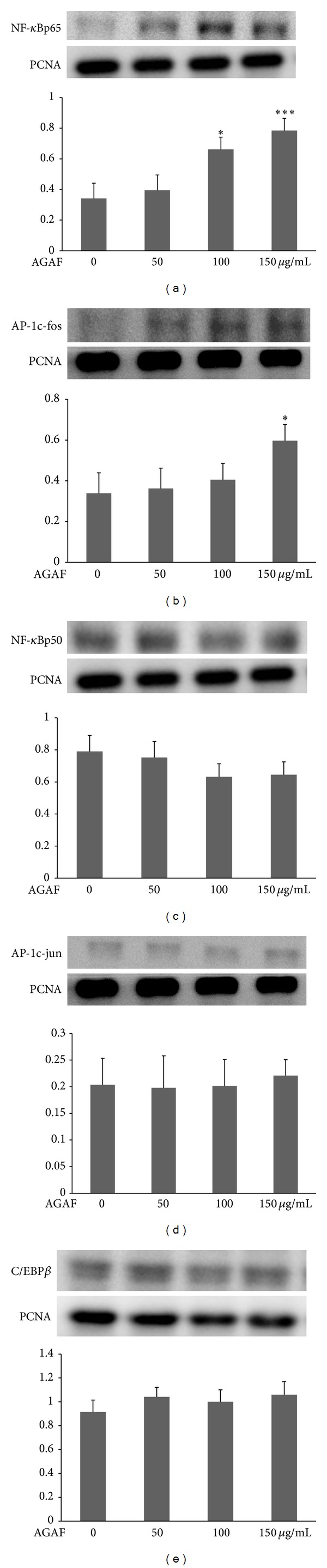
Effect of AGAF on the expression of nuclear transcription factors in RAW 264.7 cells treated with AGAF (50, 100, and 150 *μ*g/mL) or a medium for 60 min. (a) The expression of NF-*κ*Bp65, (b) AP-1c-fos, (c) NF-*κ*Bp50, (d) AP-1c-jun, and (e) C/EBP*β* was assessed via western blotting, and the expression of PCNA was used as internal. The values are presented as the mean ± SD; *n* = 3. *P < 0.05 and ***P < 0.001 as compared to control group as analyzed by Dunnett's test.

 Figures 3(a) and 3(b) showed that AGAF induced the expression of nuclei transcription factors, NF-*κ*Bp65 and AP-1c-fos. Only 150 *μ*g/mL of AGAF could induce a significant difference in the expression of AP-1c-fos. The expression of nuclei NF-*κ*Bp65 was 1.9- and 2.3-fold more than that of the control group at AGAF treatments of 100 *μ*g/mL and 150 *μ*g/mL, respectively. The expression of NF-*κ*Bp50, AP-1c-jun, and C/EBP*β* was not affected by AGAF (Figures 3(c) and 3(d)). 

### 3.3. Inhibitors of AGAF-Induced G-CSF Secretion and mRNA Expression

**Figure 4:**
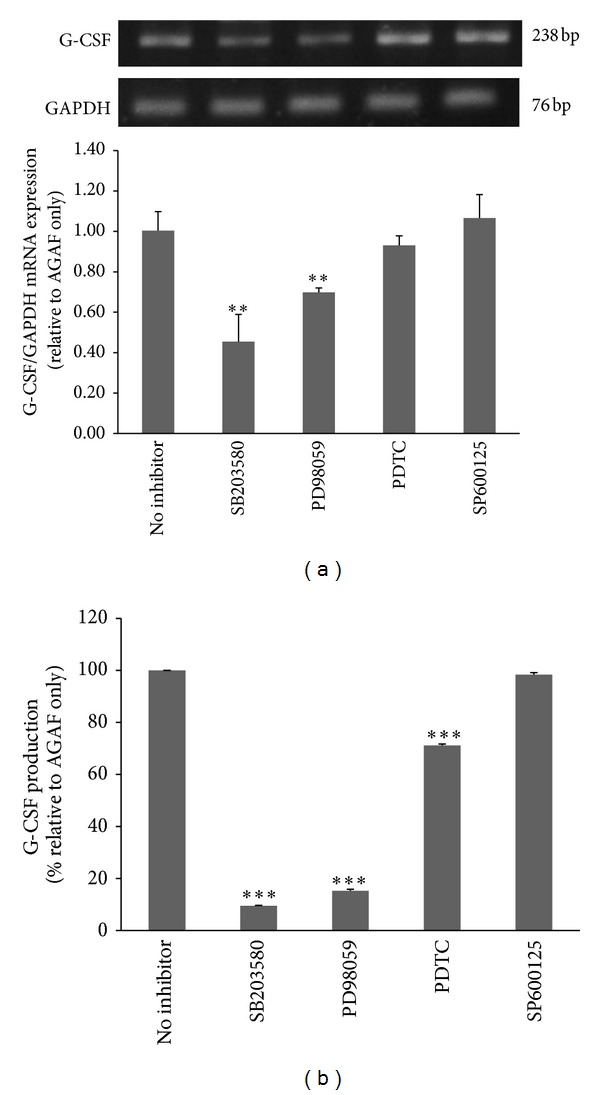
Effects of inhibitors on G-CSF mRNA expression and production in RAW 264.7 cells stimulated with AGAF (100 *μ*g/mL). (a) G-CSF mRNA expression in RAW 264.7 cells that were pretreated with specific inhibitors or a medium for 60 min and then treated with 100 *μ*g/mL of AGAF for 6 h. The G-CSF mRNA expression was examined via RT-PCR. (b) G-CSF production in RAW 264.7 cells that were pretreated with specific inhibitors or a medium for 60 min and then treated with 100 *μ*g/mL of AGAF for 16 h. The levels of G-CSF were determined by ELISA. The values are presented as the mean ± SD; *n* = 6. **P < 0.01, and ***P < 0.001 as compared to control group as analyzed by Dunnett's test.

As described, inhibitors such as SB203580 (10 *μ*M), SP600125 (10 *μ*M), PD98059 (10 *μ*M), and PDTC (25 *μ*M) were used to examine the possible signaling pathways in AGAF-induced G-CSF production. The SB203580 and PD98059 inhibitors significantly reduced AGAF-induced G-CSF mRNA expression by 54.6 ± 13.0% and 29.8 ± 2.3%, respectively (Figure 4(a)). These inhibitors also reduced AGAF-induced G-CSF production by 92.0 ± 1.6% and 91.6 ± 2.3%, respectively (Figure 4(b)). PDTC did not reduce G-CSF mRNA expression, but it significantly decreased the secretion of G-CSF by 18.2% ± 0.9%. By comparison, SP600125 did not inhibit AGAF-induced G-CSF mRNA expression or protein production (Figure 4). 

### 3.4. Analysis of NF-*κ*B Activation

**Figure 5:**
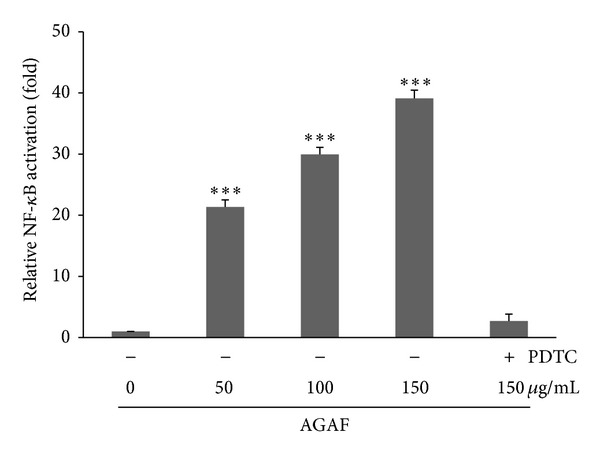
Effects of AGAF on NF-*κ*B activation were determined by luciferase assay in cultured RAW 264.7 cells. RAW 264.7 cells were treated with AGAF (50, 100, and 150 *μ*g/mL) or a medium for 24 h. 25 *μ*M of PDTC was pretreated on cells for 60 min and was used as a negative control. The values are presented as the mean ± SD; *n* = 6. ***P < 0.001 as compared to control group as analyzed by Dunnett's test.

A luciferase reporter gene assay was used to investigate the effects of AGAF on NF-*κ*B activation in RAW 264.7 cells.  Figure 5 showed that AGAF significantly induced NF-*κ*B activity in a dose-dependent manner. The NF-*κ*B activation was 21.3-, 29.9-, and 39.0-fold higher than that of the control group for AFP treatments of 50, 100, and 150 *μ*g/mL, respectively (Figure 5). In this experiment, PDTC was used as a negative control, and it markedly inhibited the AGAF- (150 *μ*g/mL)-induced NF-*κ*B activation (Figure 5).

### 3.5. Inhibition of Cytokine Production Using Pattern-Recognition Receptor Antibodies

**Figure 6:**
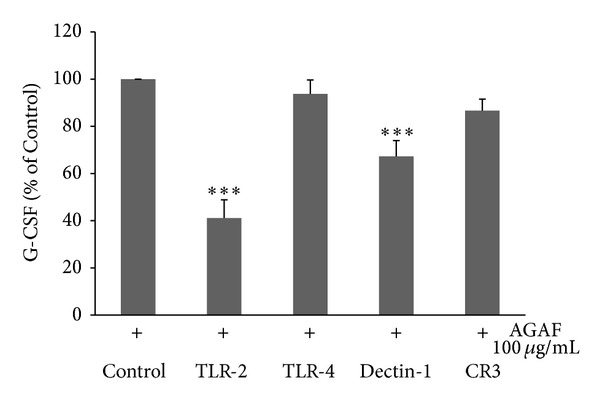
The effects of pattern-recognition receptors on G-CSF production in RAW 264.7 cells treated with AGAF (100 *μ*g/mL). RAW 264.7 cells were incubated with various function-blocking antibodies specific to TLR2 (10 *μ*g/mL), TLR4 (10 *μ*g/mL), Dectin-1 (10 *μ*g/mL), and CR3 (10 *μ*g/mL) in the presence of AGAF (100 *μ*g/mL) for 16 h. The level of G-CSF was determined by ELISA. The values are presented as the mean ± SD; *n* = 6. ***P < 0.001 as compared to control group as analyzed by Dunnett's test.

Botanic polysaccharides can active macrophages through those receptors. Therefore, this study investigated whether these receptors are involved in the AGAF-induced production of G-CSF. The results show that treatment with anti-TLR2 mAb (10 *μ*g/mL) and anti-Dectin-1 mAb (10 *μ*g/mL) significantly blocked AGAF-induced G-CSF production by 59.8% and 32.7%, respectively. Cells treated with anti-TLR4 mAb (10 *μ*g/mL) and anti-CR3 mAb (10 *μ*g/mL) failed to inhibit AGAF-induced G-CSF secretion (Figure 6). The results indicated that TLR2 and Dectin-1 might be involved in AGAF-induced G-CSF secretion. 

### 3.6. AGAF Improved the White Blood Cell Number in Mice under 5-FU Treatment

**Figure 7:**
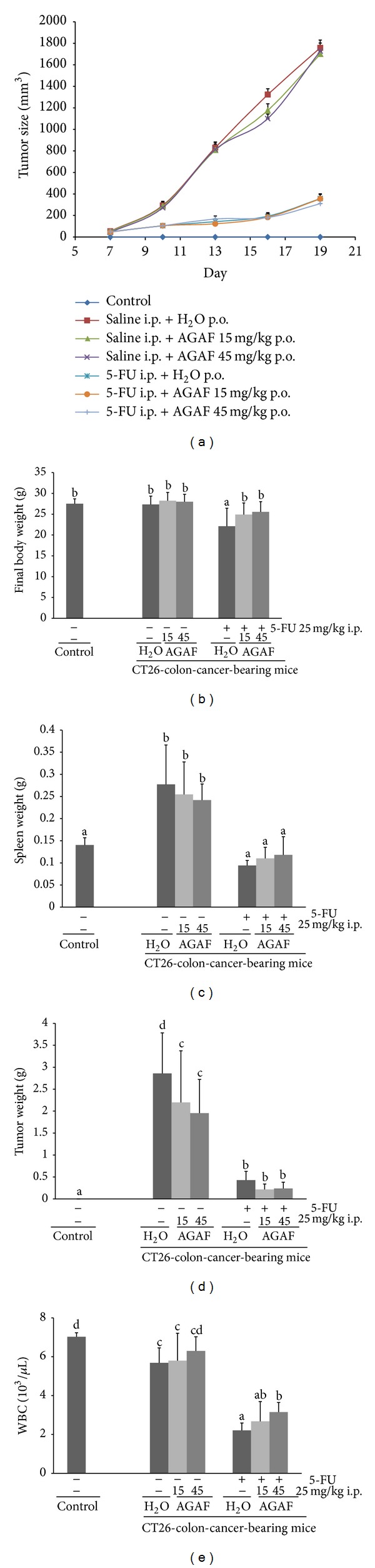
Effects of AGAF administration on CT26-colon-carcinoma-bearing mice. Mice were orally administrated with H_2_O or AGAF (15 and 45 mg/kg) every day. 5-FU treatment on mice was with 25 mg/kg i.p. every other day. Mice in control group were not inoculated with CT26. (a) The growth of tumor sizes in mice inoculated s.c. with CT26 (1 × 10^6^ cell/mouse) on Day 0. The tumor sizes were scored every three days since Day 7. (b) The final body-weight of CT26 bearing mice and control mice. (c) The spleen weight of CT26-bearing mice and control mice. (d) The tumor weight of CT26-bearing mice and control mice. (e) The number of WBC in CT26-bearing mice and control mice. The blood was analyzed under complete blood count test. The values are presented as the mean ± SD; *n* = 10. Different superscript letters denote significant differences across group as analyzed by Duncan's multiple test (*P* < 0.05).

Figure 7(a) showed that the changes of tumor size from Day 7 to Day 19 and tumor sizes decreased significantly under 5-FU treatment. However, AGAF administration could not influence the mice tumor sizes. The use of 5-FU caused a significant decrease in mice body weight compared to the H_2_O group (Figure 7(b)). The administration of AGAF (at 45 mg/kg) significantly increased body weight in mice injected with 5-FU. The injection of 5-FU reduced the spleen weight in CT26-bearing mice. Combining injection of 5-FU with the oral administration of AGAF prevented the spleen weight loss in mice (Figure 7(c)). The tumor weight of the mice orally administrated with AGAF decreased more than that of the H_2_O group. Compared to the H_2_O group, the groups treated with 5-FU had significantly reduced tumor weights (Figure 7(d)). The results of CBC testing showed that 5-FU treatment could cause a substantial decrease in the white blood cell (WBC) count; however, the administration of AGAF (at 45 mg/kg) could reduce the leukopenia caused by 5-FU (Figure 7(e)). 

## 4. Discussion

AGAF, a type II arabinogalactan isolated from *Anoectochilus formosanus*, was used in this study to investigate the stimulation and signaling pathway of G-CSF in vitro and the haemopoiesis effects on chemotherapy-treated mice. Arabinogalactans are classified into 2 structural types [25]. Arabinogalactans with a (1→4)-linked galactan backbone are classified as type I arabinogalactans [25, 26]. Type I arabinogalactans are usually linear and found in pectic complexes, such as potato [25]. Type II arabinogalactans have a (1→3; 1→6)-linked galactan backbone and are more widely distributed in plants than type I arabinogalactans [25]. Type II arabinogalactans extracted from plants are said to exhibit a number of bioactivities, especially in immuno-stimulation [27, 28]. This study demonstrates the effects of AGAF, a type II arabinogalactan, on G-CSF secretion and haemopoiesis in vitro and in vivo. 

G-CSF is a member of the glycoprotein growth factor family that affects the survival, proliferation, and differentiation of hematopoietic cells [29]. The production of G-CSF is not constitutive and can be induced by a wide variety of stimulatory agents, including LPS, TNF-*α*, IL-1*β*, phorbol 12-myristate 13-acetate, and IFN-*γ* [11]. Polysaccharides are a group of PAMPs. They regulate the PRRs that can induce a series of immune responses, creating an effective defense against distinct pathogens. In this study, AGAF functioned as a PRR to stimulate G-CSF production in murine macrophages. The secretion of G-CSF is the prophase immunity response that could further affect neutrophil proliferation. Neutrophils and macrophages are phagocytes whose principal function is to maintain host-defense in innate immunity. Neutrophils can ingest and kill invading bacteria, releasing cytotoxic, chemotactic, and inflammatory mediators at the infection sites in an immediate host immune response. In addition, neutrophils are regulated by G-CSF, the principal cytokine controlling neutrophil development and function [30]. The ability to produce G-CSF is characteristic of various cell types following appropriate stimulation. Cells of the monocyte/macrophage lineage represent major sources of G-CSF [11]. Accordingly, a murine macrophage cell line, RAW 264.7, was used as a cell model in this study.

The results of this study showed that AGAF significantly induced G-CSF in RAW 264.7 cells. However, the LPS contamination in AGAF may induce the expression of G-CSF in RAW 264.7 cells [31]. The ability of AGAF to induce G-CSF secretion may be the result of LPS pollution. Therefore, AGAF was analyzed for endotoxin contamination using a Limulus amebocyte lysate assay, revealing less than 0.01 EU/mL. This indicated that the induction of G-CSF secretion is not attributable to LPS pollution. 

**Figure 8:**
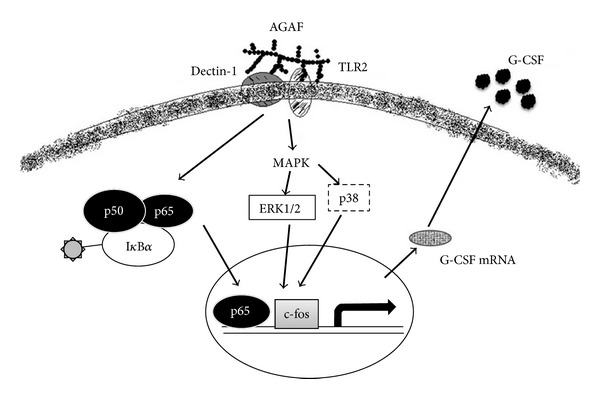
The schematic model illustrating potential signaling pathways triggered by the proposed binding event of AGAF with TLR2 and Dectin-1 in G-CSF production.

The p38-MAPK (SB203580), ERK-MAPK (PD98059), and NF-*κ*B (PDTC) inhibitors clearly reduced AGAF-induced G-CSF secretion. In addition, AGAF induced the phosphorylation of p38, ERK1/2, and I*κ*B*α*, but not JNKs. However, the JNK (SP600125) inhibitor did not block AGAF-induced G-CSF protein production. These results suggest that AGAF induces G-CSF expression through the MAPK and NF-*κ*B pathways (Figure 8). In this study, the p38 inhibitor inhibited G-CSF production the most. These results indicate that the p38-MAPK pathway is related to G-CSF production. In mammalian cells, p38-MAPK plays a central role in the regulation of various inflammatory responses, including the expression of proinflammatory mediators, leukocyte adhesion, chemotaxis, oxidative burst, and degranulation [32]. Previous research on the signaling protein activation and cytokine release of RAW 264.7 cells has shown that the p38 MAP kinase pathway also plays an essential role in G-CSF production [31]. 

The ERK1/2 inhibitor could significantly reduce AGAF-induced G-CSF, suggesting that ERK1/2 might be involved in the G-CSF production of AGAF. Furthermore, ERK signaling is a central MAPK pathway that plays regulatory roles in various cellular processes, including proliferation, differentiation, development, learning, survival, and, under some conditions, apoptosis [33]. 

Three conserved upstream regions have been identified in the G-CSF promoter, including binding sites for the octamer, NF-*κ*Bp65, and a CCAAT enhancer-binding protein beta (C/EBP*β*). The remaining 2 regions are required for the induction of the gene [31, 34]. The binding of NF-*κ*B and C/EBP*β* in the G-CSF promoter might mediate the transcriptional activation of G-CSF [35, 36]. The results show that PDTC suppressed the AGAF-stimulated release of G-CSF from RAW 264.7 cells, suggesting that NF-*κ*B is a key transcription factor involved in AGAF-induced G-CSF expression. In addition, AGAF induced the NF-*κ*B activation in the luciferase assay in a dose-dependent manner. In the nuclei transcription factor expressions analyses using a western blot, AGAF induced the content of the transcription factor NF-*κ*Bp65, but not C/EBP*β*. These results indicated that C/EBP*β* was not involved in the pathway of the AGAF-induced G-CSF secretion. In addition, JNK may contribute to the transcriptional activation of C/EBP*β* in macrophages [37]. In this study, the un-raised level of C/EBP*β* may result from the inactive expression of JNKs in macrophages under AGAF treatment.

The expression of the G-CSF gene is regulated by a combination of transcriptional and posttranscriptional mechanisms [11]. In this study, NF-*κ*Bp65 was involved in the transcriptional factor, and the activation of p38 might have been related to posttranscriptional regulation. Previous research has shown that G-CSF mRNA contains AU-rich destabilizing elements (AREs) in the 3′-untranslated region [38], and recent evidence has suggested that the p38 pathway plays a role in the regulation of ARE mRNA stability [39]. 

Macrophage activation by plant polysaccharides is likely mediated by the recognition of polysaccharide polymers by specific receptors [9]. These receptors are pattern-recognition molecules that can recognize foreign ligands during the initial phases of an immune response [40]. Specifically, macrophages might bind botanical polysaccharides through TLR2, TLR4, CD14, CR3, Dectin-1, scavenger receptor, and mannose receptor [9]. Pradervand et al. [31] used a RAW 264.7 model and found that, among the Toll-like receptors, TLR2/1, TLR2/6, TLR4, and TLR7 are involved in the signaling networks leading to G-CSF release. The results of this study show that AGAF-TLR2 binding primarily affects AGAF-induced G-CSF production (59.8% in inhibition). When plant polysaccharides act on receptors, several receptor types are likely to cooperate with each other (e.g., TLR4-CD14, Dectin-1-TLR2, or CR3-CD14) to form clusters of signaling complexes [9, 41]. In this study, it was found that the Dectin-1 mAb reduced the production of G-CSF in the inhibition of 32.7%. These results suggest that macrophages may recognize AGAF through TLR2 and Dectin-1 receptors (Figure 8). Dectin-1, which is a *β*-glucan receptor, and TLR2 could be stimulated with *β*-glucans, similar to yeast zymosan [42]. However, in this study, TLR2 and Dectin-1 recognized AGAF, a type II arabinogalactan. It did not come as a surprise that, although AGAF is not a *β*-glucan, it acted on the *β*-glucan receptors, TLR2 and Dectin-1. Previous research has reported that Dectin-1 acts with TLR2 to mediate macrophage activation with mycobacteria. In addition, mycobacteria-infected macrophages produced G-CSF. The mycobacteria cell walls containing mycolyl arabinogalactan peptidoglycan are identified as factors responsible for inducing tissue factors in macrophages. This may be the reason that AGAF, an arabinogalactan isolated from plants, could also be recognized by TLR2 and Dectin-1 in macrophages. Krestin, a TLR2 agonist polysaccharide isolated from mushrooms, mediates the inhibition of tumor growth through immune stimulation [43]. Lu et al. [43] showed that TLR2 is involved in the antitumor activity of Krestin and demonstrated this in WT and TLR2^−/−^ mice. 

The relevance of G-CSF to the immune response has been demonstrated in previous studies, showing that G-CSF has a beneficial effect on the outcome of infection [11]. G-CSF is used to prevent neutropenia in patients with solid tumors who are receiving chemotherapy [44]. CT26 cells are mouse colon carcinoma cell lines and are subcutaneously inoculated to BALB/c mice to establish a solid cancer model. The 5-FU chemotherapy drug has been used against cancer for approximately 40 years [45]. 5-FU has many adverse drug reactions (ADRs) such as diarrhea, stomatitis, nausea, weight loss, and leukopenia. Among these ADRs of 5-FU, severe leukopenia may lead to potentially hazardous delays in treatment and life-threatening bacterial and fungal infections [20]. This study showed that the support of AGAF-induced G-CSF may reduce the leukopenia on CT26-inoculated mice injected with 5-FU. The results indicated that oral administration of AGAF reduced the loss of body weight and increased the number of WBC in CT26-inoculated mice treated with 5-FU. The oral administration of AGAF could decrease the tumor weight compared to the H_2_O group; however, the treatment combining AGAF and 5-FU could not reduce the tumor weight more than the 5-FU group could. Although the combined treatment did not achieve a higher improvement in tumor size, supplementing AGAF could reduce the ADR during 5-FU treatment. 

## 5. Conclusion

In summary, this paper provided the first evidence that AGAF increases G-CSF secretion in RAW 264.7 cells and demonstrated that intracellular signaling is involved in AGAF-induced G-CSF secretion through the activation of MAPK and NF-*κ*B signaling pathways. The supplement of AGAF reduced the leukopenia caused by 5-FU injection in CT26-carcinoma-inoculated mice.
